# Older patients’ perspectives on factors contributing to frequent visits to the emergency department: a qualitative interview study

**DOI:** 10.1186/s12889-021-11755-z

**Published:** 2021-09-20

**Authors:** Daisy Kolk, Anton F. Kruiswijk, Janet L. MacNeil-Vroomen, Milan L. Ridderikhof, Bianca M. Buurman

**Affiliations:** 1 Amsterdam UMC, University of Amsterdam, Emergency Medicine, Amsterdam Movement Sciences, Meibergdreef 9, Amsterdam, Netherlands; 2 Internal Medicine, Section of Geriatric Medicine, Amsterdam UMC, University of Amsterdam, Internal Medicine, Section of Geriatric Medicine, Amsterdam Public Health, Meibergdreef 9, Amsterdam, Netherlands; 3 OLVG Hospital, Department of Geriatric Medicine, Amsterdam, the Netherlands; 4 ACHIEVE - Centre of Applied Research, Faculty of Health, Amsterdam University of Applied Sciences, Amsterdam, Netherlands

**Keywords:** Patient experiences, Aged, Geriatrics, Qualitative research, Acute care

## Abstract

**Background:**

Older patients are at high risk of unplanned revisits to the emergency department (ED) because of their medical complexity. To reduce the number of ED visits, we need more knowledge about the patient-level, environmental, and healthcare factors involved. The aim of this study was to describe older patients’ perspectives and experiences before and after an ED visit, and to identify factors that possibly contribute to frequent ED revisits.

**Methods:**

This was a qualitative description study. We performed semi-structured individual interviews with older patients who frequently visited the ED and were discharged home after an acute visit. Patients were enrolled in the ED of a university medical centre using purposive sampling. Interviews were recorded, transcribed, and coded independently by two researchers. Theoretical analysis was used to identify recurring patterns and themes in the data. Interviews were conducted until thematic saturation was reached.

**Results:**

In-depth interviews were completed with 13 older patients. Three main themes emerged: 1) medical events leading to feelings of crisis, 2) patients’ untreated health problems, and 3) persistent problems in health and daily functioning post discharge. Participants identified problems before and after their ED visit that possibly contributed to further ED visits. These problems included increasing symptoms leading to feelings of crisis, the relationship with the general practitioner, incomplete discharge information at the ED, and inadequate follow-up and lack of recovery after an ED visit.

**Conclusions:**

This qualitative study identified multiple factors that may contribute to frequent ED visits among older patients. Older patients in need of acute care might benefit from hospital-at-home interventions, or acute care provided by geriatric emergency teams in the primary care setting. Identifying frailty in the ED is needed to improve discharge communication and adequate follow-up is needed to improve recovery after an acute ED visit.

**Supplementary Information:**

The online version contains supplementary material available at 10.1186/s12889-021-11755-z.

## Background

More than 18% of all emergency department (ED) visitors in the United States, like many other countries, are older than 65 years [1] and the number of older patients presenting to overcrowded EDs is increasing [2, 3]. Older patients that frequently visit the ED have multiple chronic conditions, more severe illness, and more complex care questions [3–7]. Moreover, older patients are at high risk of unplanned revisits; more than 50% are discharged home from the ED [6, 8] and approximately 10 to 23% have to return unexpectedly within the first month [5, 6, 9].

The complex care needs and unique challenges of older patients presenting to the ED often involve geriatric syndromes [9]. Geriatric syndromes like cognitive and functional impairment, falls, and malnutrition are highly prevalent among older patients [9, 10] and may explain the need for frequent visits to the ED. However, geriatric syndromes often remain undiagnosed or undertreated, which increases the need for further ED visits [9].

The development of effective interventions to reduce ED visits among older patients is challenging [11]. Interventions focusing on discharge planning [12], transitional care [13], and phone calls after discharge [14] have not effectively reduced ED revisits. Developing effective interventions to prevent unplanned ED visits requires in-depth knowledge of patient-related, environmental, and healthcare-related factors. Given the complexity of these factors and the interaction between them, a qualitative approach is well suited for exploring this phenomenon.

The aim of this study was to describe the perspectives and experiences of older patients before and after a visit to the ED and to identify why these patients may have to return unexpectedly to the ED.

## Methods

We utilized the Standards for Reporting of Qualitative Research [15], the criteria for reporting qualitative research (COREQ) [16], and the best practice guidelines to generate and report our findings [17, 18].

### Study design

We performed a qualitative description study [19], to provide a rich description of older patients’ perspectives and experiences before and after their visit to the ED and to identify possible contributing factors to unplanned revisits. This inductive approach is suitable for problem identification and hypothesis generation and is especially useful for research questions in health care because it helps to focus on the patients’ experiences and views on the health care system [20]. This method aims to provide a rich, straight description of perceptions and experiences and is founded in existing knowledge and clinical experiences of the research group, instead of other qualitative methods that are theory-driven [20, 21]. We conducted semi-structured individual interviews which allow for a detailed in-depth exploration of the patient’s perceptions and experiences [22].

### Study setting and population

Between June 2019 and September 2019 this study was conducted in the ED of a University Hospital’s Level I trauma center in the Netherlands, treating approximately 30.000 patients annually. The hospital had an accredited residency program in Emergency Medicine and the department is staffed by fully trained Emergency Physicians 24/7. When required, consultants of all medical specialties are available, including geriatrics. In this ED, approximately 46% of the older patients are discharged home by the treating physician, who decides what type of after care is needed (e.g., follow-up consultation; referral to the general practitioner or to an outpatient clinic).

Older patients (≥ 70 years) who frequently visited the ED and were discharged home after their last visit (the index visit) were eligible for inclusion. Further inclusion criteria were a medical history of two or more morbidities, and a previous visit to the ED or hospital during the past 18 months. Patients were ineligible if they were not able to speak Dutch sufficiently to perform the interview or were not able to give informed consent or perform the interview due to moderate/severe cognitive impairment judged by the treating physician. We used a purposive sampling method to identify relevant patients to interview, and to reach maximum variation in heterogeneity within this population regarding age, admission diagnosis, treating physician, and living situation [23]. All participants gave informed consent to take part in the study. The Institutional Review Board waived the need for approval under the Medical Research Involving Human Subjects Act.

### Study protocol

One of two researchers (DK and AK) recruited participants and performed the interviews. DK is a clinical epidemiologist and PhD student in the department of Emergency Medicine and Geriatrics with formal qualitative research training. AK is a physician and worked as a research assistant in the ED. DK trained AK in qualitative research techniques. Neither interviewer was part of the participants’ medical care team.

Patients were recruited at the ED prior to or immediately after the discharge conversation. Patients were screened for eligibility by the treating physician and eligible patients were contacted by the study staff. The researcher informed the patient about the study, answered questions, ensured that the patient had comprehended the information and obtained informed consent. The researcher collected basic demographic information at the ED and an appointment for the interview was made. To minimize recall bias, interviews were conducted between 7 and 30 days after discharge [24]. Interviews lasted approximately 90 min and took place face-to-face at the participant’s home to ensure their privacy and comfort. Family members or informal caregivers of the participant were allowed to participate.

The interview guide consisted of a topic list and open-ended questions that were formulated based on a literature search and (clinical) experiences of senior researchers, emergency physicians, geriatricians, and nurses. The complete interview guide is shown is an additional file (see Additional file 1). The interview guide was pilot-tested and was iteratively revised during the interview process. All interviews were voice-recorded and transcribed verbatim by the research staff. During the interview the researcher took field notes for recall of the context. Given this specific population, transcripts were not returned to the participants and participants were not asked to provide feedback on the findings afterwards, but the interview was verbally summarized and discussed immediately with the participant after the interview.

### Data analysis

We used theoretical analysis, a type of thematic analysis used when the researcher has some pre-understanding of the topic, to identify, analyse and report patterns in the data and formulate themes [25]. DK and AK independently generated initial themes using an inductive open-coding approach, by highlighting meaningful sentences in the text and coding all relevant topics. After the initial coding, DK and AK reread all coded data to identify patterns. Patterns were compared between interviews to reach a conclusion about main themes throughout the interviews. During the analysis, we remained open to the possibility of new categories coming up, and thoughts and changes in the coding scheme were discussed thoroughly within the research team. Finally, a list of relevant main themes with subthemes was created and the results were described in detail, illustrated with extracts from the transcripts. We conducted interviews until theoretical data saturation was reached [26]. The Computer Assisted Qualitative Data Analysis program (MaxQDA) was used to code and manage the qualitative data [27]. During this process, memo’s and manual version control were used to increase the auditability.

## Results

Thirteen participants completed the interviews, and thematic saturation was reached after nine interviews. Thirty-two older patients were screened for eligibility; 23 were eligible and asked to participate. Four patients were not interested in research in general and two felt too ill to give informed consent. Seventeen older patients gave informed consent, but eventually four of these patients felt too ill to be interviewed and withdrew their informed consent. Table 1 presents the characteristics of the 13 participants. They had a mean (SD) age of 75 (6), ranging from 70 to 91 years, a median (IQR) number of 4 (3–4.5) comorbidities, and 7 (54%) participants had their informal caregiver involved in the interview.

**Table 1:** Participant Characteristics (*n* = 13)

Variables ^**a**^	
Sex, female	7 (54)
Age (years)	
Mean (SD)	75 (6)
Range	70–91
Race	
Asian	2 (15)
White	11 (85)
Marital status	
Living with partner	8 (62)
Widowed	3 (23)
Divorced/single	2 (15)
Number of comorbidities ^b^	
Median (IQR)	4 (3–4.5)
Cancer diagnosis	4 (30)
Severe hearing or vision impairment	2 (15)
Experienced a fall in the past 6 months	2 (15)
Functional dependence ^c^	4 (30)
Informal caregiver involved in the interview	7 (54)

SD, standard deviation; IQR, interquartile range; ED, emergency department.

^a^ All variables are presented in a number and percentage unless otherwise indicated

^b^ Collected from the hospital medical records

^c^ Score of ≥1 from the six-item Katz-ADL

**Fig. 1:**
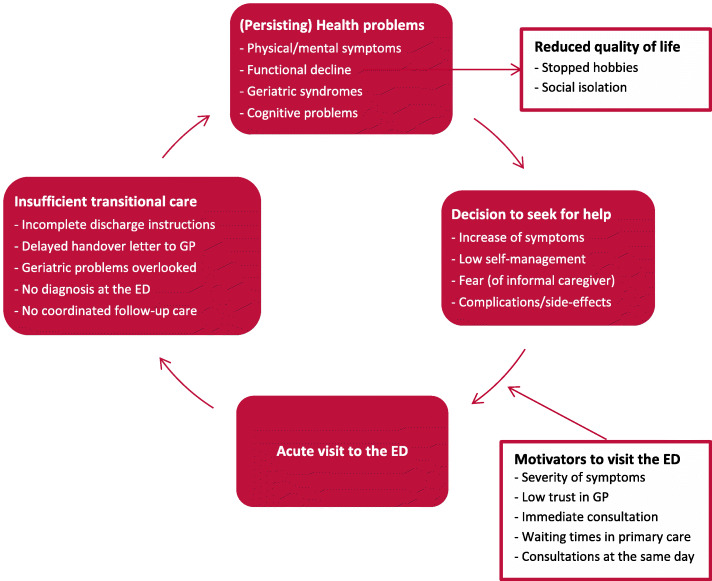
Possible contributing factors to frequent ED visits based on reported patient experiences. ED, emergency department; GP, general practitioner

Based on the thematic analysis of the interviews, three main themes emerged: 1) medical events leading to feelings of crisis; 2) patients’ untreated health problems; and 3) persistent problems in health and daily functioning post discharge. Table 2 summarizes the themes and provides illustrative quotes. Figure 1 summarizes the conceptual model that was constructed based on the identified themes.

**Table 2:** Summary of themes and illustrative quotes

Themes	Illustrative quotes
Theme 1: Medical events leading to feelings of crisis	
1.1 Decision to seek medical care	My brother called me unexpectedly to come over and I saw that things were not going well with my mother. We had to go to the ED because there was nothing we could do about her lung condition and the ED was our only option. (Informal caregiver of participant 5; female, 70)
1.2 Motivators to visit the ED	I called the gastroenterologist who said, “You have to go to the ED as that is the fastest and the best solution.” And so I went. (Participant 6; male, 70)
Theme 2: Patients’ untreated health problems	
2.1 Discharge from the ED	“The doctor will be with you shortly.” Well, minutes turned into hours and finally the doctor appeared. “Now I saw the X-rays and your collar bone is broken, so you may go home now.” I got a sling and that is it. (Participant 11; male, 79)
	However, maybe you should put it on paper. We had a whole conversation with the doctor and the doctor said, “You must do this, you must do that.” Then there was so much information that came at me. And some things I just forgot. That is … and if it was on paper, I could refer to what was said. What were the action points that needed to happen? (Informal caregiver of participant 5; female, 70)
2.2 Follow-up and continuity of care	We are disappointed. We did not realize that we were responsible for organizing the follow-up appointment with the outpatient clinic. We thought the ED would do that. (Participant 3; female, 73)
Theme 3: Persisting problems in health and daily functioning post discharge	
3.1 Physical and mental symptoms	It is a bit weird and crazy, but I do not dare to go outside! I am too afraid, but I do not understand why. I panic at the thought of it. However, if I went upstairs, then I did not remember anymore why I was going there. It was probably my own insecurity, I was confused, and I just did not remember things well. That was really disturbing and worrying. (Participant 12; female, 82)
3.2 Effects on daily life	I cannot do anything. No, I can walk a bit. I sit a lot. Vacuuming, cleaning, that sort of things, I cannot do that. Yes of course, I am not happy about it. I do not see any progression in my recovery and I expected that. Because, until now … so far things are not so positive. (Participant 6; male, 70)

ED, emergency department

### Theme 1 – medical events leading to feeling of crisis

We identified two categories that came up in most interviews regarding events leading up to the index visit: 1) the decision to seek medical care, and 2) motivators to visit the ED.

#### Decision to seek medical care

In most participants, health problems and symptoms were already present for several weeks or months before the current ED visit. Some of them had visited the ED recurrently for the same symptoms before the index visit. Some participants said that the index visit was a result of escalation as they suddenly experienced an increase in severity of symptoms for a longer period, as participant 1 (female, 71) recounted: “So first I was just waiting and then I was just thinking whether this would take care of itself or would these symptoms persist? I wanted to wait two weeks, but then I just felt so sick. So then I just thought I will just call the doctor [medical specialist].” Or participants or their informal caregivers were afraid of an increase in symptom severity, or they did not know how to handle the symptoms anymore. They thought it was inevitable to present to the ED. This was true for participant 5 (female, 70), who recounted, “It is getting so bad, I really don’t know what to do. I have never been so sick and used so much medication. I really panicked: what is happening to me?” In other cases, participants decided to visit the ED after an unexpected acute situation, like a fall, as participant 8 (male, 91) said, “I have had falls, but not as bad as this. There was a puddle of blood, and it was not stopping because of my blood thinners.” Some participants described that they were clearly instructed by their treating medical specialist to go to the ED if a particular symptoms occurred after a medical intervention. In some cases, this occurred several times. For example, participant 12 (female, 82) recounted, “I was discharged home and instructed to come back if I had a fever. So, we went to the ED when I had a fever, but they could not find anything. So I was sent home and the fever returned. I went back to the ED several times as the doctor told me to.”

#### Motivators to visit the ED

Participants most frequently mentioned logistic or organizational aspects in their decision to visit the ED instead of initially referring to other healthcare services. There were many underlying thoughts and opinions related to their situations. Some participants decided to visit the ED by themselves, because they experienced before that they needed specific diagnostics after a fall. For example, participant 11 recounted: “By every fall, you never know what you have. You never know if something is broken, so you have to go to the ED to get X-rays taken.” In other cases, participants experienced symptoms for a longer period but decided to visit the ED as they thought waiting times in the outpatient clinic or general practice would be too long for consultation and diagnostics. Even though they had waited a long time at the ED before, they still went there because they wanted a consultation including ancillary testing on the same day. Some participants who were vulnerable or had a small social network said that visiting the ED once was less of a burden than visiting the outpatient clinic several times. For example, participant 3 (female, 73) had experienced symptoms for a long time and was not in good physical condition. Her informal caregiver recounted, “Yes, I called the outpatient clinic to talk to her treating medical specialist. And he advised us that it was more efficient to go to the ED, otherwise we would have to make several outpatient appointments to get same care and that would be far too burdensome.”

When asked why they visited the ED, many participants mentioned their relationship with their general practitioner (GP) and recounted a wide array of experiences. Many patients described that they had a good relationship with their GP but felt that they needed the expert opinion of their medical specialist because of their particular conditions. Others described past experiences that diminished their confidence in their GP, such as participant 5 (female, 70), who said, “I don’t expect anything from my new GP. When I see the GP, I sit down, I say what the problem is; and then nothing happens. I would like to have new one, but the waiting lists are too long.”

### Theme 2 – patients’ untreated health problems

Two categories emerged under this theme: 1) discharge from the ED, and 2) follow-up and continuity of care.

#### Discharge from the ED

The majority of the participants indicated that they were happy to be discharged home after often a whole day in the ED. Many participants felt stressed at the time of the ED visit, and could not remember all the details after being discharged. In particular, those who did not receive a clear diagnosis stated that they would like to talk about their health problems some days after the ED visit, when they were less stressed. Some participants described visiting the ED with severe symptoms and being discharged without a diagnosis. They felt temporarily reassured, but then felt insecure back at home because their symptoms were unresolved. These patients felt frustrated because they did not receive further information at discharge, as participant 3 (female, 72) described: “They [ED doctors] didn’t say that much. I do not even know if I got a discharge letter. No, I did not! All they said was ‘Madam, we did not find anything wrong with you.’ Then they said, ‘Your GP will do further follow-up.’ Or maybe they said the medical specialist.”

Some participants felt the focus in the ED was solely on somatic care, even though they also needed psychological support and practical help. Participant 8 (male, 91) said that he was alone in the ED and was told he would be discharged in the late evening. He felt unsatisfied because he did not receive any help arranging his discharge. Many participants felt the information at discharge was incomplete. One informal caregiver described that they received a lot of verbal information, but could not remember it all after discharge. This informal caregiver said that written information at discharge would have been more helpful and would have reduced the stress they felt in the ED at not understanding everything they were told. This was less of a problem for the two participants who visited the ED with cancer-related medical problems. They said they received very good cancer-related care in specific care pathways. Because they were already familiar with their diagnosis and had follow-up appointments with their medical specialist, they felt less need for a comprehensive conversation at discharge. For example, participant 6 (male, 70) recounted: “I did have meetings with the palliative care team, and they called again today. Together with the palliative care team, my GP, and my oncologist in the hospital. As I already had regularly scheduled check-ups, an appointment for follow-up care after my ED admission was not necessary. I did not need any comprehensive discharge instructions as I know how everything works already.”

#### Follow-up and continuity of care

Many participants said that a follow-up appointment was quickly arranged after discharge. They felt reassured by their post-discharge appointment and the medical follow-up it provided. Many participants felt the need for more information and had many questions for their physician. In cases where follow-up was not arranged, patients felt stressed or unsafe. Moreover, participants also felt they needed to participate actively in their own care, as the informal caregiver of participant 4 (female, 72) explained: “You really need to think for yourself; otherwise something could be forgotten or missed in your patient file. But you have to figure that out yourself the hard way. If you are cognitively impaired and are on your own, then you are really vulnerable.” Some participants also said that they called their physician soon after discharge when symptoms increased. One participant described that she went to see her GP, but was referred back to the ED because the GP had not received the discharge letter after the last ED visit.

Most patients with a diagnosis of cancer said that a follow-up appointment with their medical specialist was already planned before the index visit. They often felt that their frequent visits to the ED were minor events in their declining health and were part of their illness. Participant 9 (female, 72 years) stated: “A visit to the ED is an accepted part of the process in my declining health. With cancer it is inevitable.”

### Theme 3 – persistent problems in health and daily functioning post discharge

Within this theme, two categories emerged: 1) physical and mental symptoms, and 2) effects on daily life.

#### Physical and mental symptoms

All participants described that increasing symptoms negatively affected their physical and mental health. Some participants felt that the index visit did not resolve their current health problems, and usually ended up back at the ED, where they did not receive a diagnosis. This resulted in persisting symptoms and concern, unless serious diseases were excluded. Participant 1 (female, 71) recalled: “After the ED visit, I thought to myself ‘I still have the same symptoms.’ Then I started to second-guess the advice of the ED and I wondered if they had missed something. And then I really started to worry and just kept on worrying.”

After they were discharged from the ED, participants experienced problems with recovery and reported several physical and psychological problems. Most of these health problems were already present prior to the ED visit, however, more than half of the participants mentioned new problems that occurred after leaving the ED. When asked about their problems, they mentioned several symptoms like decreased appetite, loss of muscle strength, fear of falling, and fatigue. Participant 8 (male, 91) described his recovery after his last ED visit: “I know I really must eat, as I already lost 10 pounds. I am doing the best I can. In the morning and in the afternoons, I have more appetite. However, at dinnertime I just cannot eat. I am afraid to fall if I go outside. I always take my cane to go to the garage. It is not easy, but it gives me some security.”

#### Effects on daily life

The majority of participants described that they experienced problems in their daily functioning and were not able to resume all their usual activities because of their health problems. They recounted a decline in functioning before their recent ED visit, for example after a previous hospital admission. Many participants felt that the index visit had not improved their current health problems nor their functionality. They were disappointed because they expected their symptoms to decrease and their physical functioning to increase. Participant 6 (male, 70) described: “I cannot do anything. No, I can walk a bit. I sit a lot. Vacuuming, cleaning, that sort of things, I cannot do that. Yes of course, I am not happy about it. I do not see any progression in my recovery, and I expected that. Because, until now … so far things are not so positive.” Some participants believed that ageing and their declining health were responsible for their frequent ED visits and functional decline, as participant 8 (male, 91) described: “That’s what happens when you get older. It is like a snowman, you just melt away with time and that is hard to accept. You just keep on declining.”

Some participants felt that their symptoms and functional decline affected their quality of life. For example, the informal caregiver of participant 5 (female, 70) recounted that his mother was not able to go outside anymore because she had lost functional ability, resulting in social isolation which negatively affected her mental health. The informal caregiver said: “I think it would be better for her to move to a place with an elevator and where she can use a scooter or something. Then it is easier for her to go outside and to the local city centre. Now she is stuck inside worrying the entire day. She would be happier if she could go outside and find some nice distractions.”

Many participants also recounted that frequent hospital visits caused a lot of stress and energy loss. Older informal caregivers described that ED visits caused an overload as they had to arrange everything unexpectedly in a very short time and they felt very insecure. Participant 12 (female, 82) recounted that she and her partner gave up their hobby after so many stressful hospital visits: “We gave up Nordic walking even though we love it. With all those ED visits, we are exhausted and it is too difficult for my husband. We have been so many times to the hospital for ED and outpatient visits. We are just so burnt out from it all.”

## Discussion

Through in-depth interviews, we identified three major themes related to older adults’ experiences before and after an ED visit: 1) medical events leading to feelings of crisis, 2) patients’ untreated health problems, and 3) persisting health problems in health and daily functioning post discharge. This in-depth description of the events leading up to an ED visit and the lack of recovery after discharge highlights potential reasons for ED revisits. These findings may improve future interventions in older patients who frequently visit the ED, and may help reduce the number of ED revisits.

Before their ED visit, most patients were experiencing symptoms that had been present for several weeks or months. These symptoms suddenly increased in severity, causing anxiety and compelling the patients to seek urgent help [28]. They felt that they were not able to control their symptoms and could no longer manage their situation, so made an acute visit to the ED. In some older patients, lower levels of self-efficacy and self-management seemed to play a role, which is a major problem among older patients with multiple chronic illnesses [29]. These results suggest that the escalation of symptoms before the ED visit could have been prevented if the patients’ symptoms were managed earlier on. When asked about ways to prevent new ED visits, some patients mentioned that they trusted the care in the ED more than they trusted the care from their GP. Trust in a very important component of the doctor-patient relationship [30] and previous studies have shown that a good relationship with the GP and greater continuity of primary care reduces ED visits in older patients [31–33]. An important determinant of older patients’ trust in the GP is the sense of shared-decision making [34]. In the Netherlands, the GP holds a central role in primary healthcare including care for older patients [35]. Therefore, it is especially important to educate GPs on the complex care needs of older patients [36]. However, older patients often need different healthcare services [2], so case management and hospital care at home would help GPs to reduce ED visits. For example, specialized geriatric medical emergency teams with access to diagnostics could provide high-quality care to patients in their own home, thereby reducing the need for ED visits [37].

After treatment in the ED, all patients included in this study were discharged home. They all agreed to be discharged, but found it very difficult to manage the transition to home life. They found their ED visit stressful [38] and did not receive the information they needed at discharge [39]. Especially in older patients with limited health literacy, incomplete information at discharge may not meet the patient’s needs [40]. Our participants reported that care in the ED was focused on somatic treatment, and failed to meet all of their needs. Previously, it has been suggested that current disease-oriented and episodic models of emergency care do not adequately meet the complex care needs of frail older patients [2]. Identifying frailty in the ED is a major problem in caring for older adults [41], but is important for a fully informative discharge and for adequate follow-up to prevent further decline [11, 41, 42]. For example, any cognitive impairment should be stated in the discharge letter because it increases the chance of readmission after discharge [43]. All physicians working with older patients in the ED need to be trained in geriatric competencies [44, 45], and older patients that frequently visit the ED should consult a geriatrician [44, 46]. Moreover, trained nurses specializing in the complex care needs of older patients may improve care transitions, effectively reducing functional decline and hospital admissions [47, 48].

Older patients who were discharged home without a specific diagnosis experienced physical and mental problems that persisted or even increased. These patients felt reassured at discharge but started worrying when they were back home and needed proper follow-up. Moreover, many of these patients experienced common post-hospital symptoms after discharge [10, 49]. Most patients had experienced functional decline before visiting the ED, and did not feel they had recovered to their baseline level after discharge. Older patients have a high risk of functional decline after being discharged from the ED [8, 50], and more than half of older patients need help with rehabilitation after discharge [51]. Based on what our participants described, we concluded that patients were passively waiting for recovery and did not consider asking for help with rehabilitation. However, a previous qualitative study [38] showed that older patients who seek emergency care have a strong desire for functional recovery. They expected that their functional difficulties would be addressed in the ED, but realized at discharge that the ED was not the right place for improving functionality and health-related quality of life. These findings are in line with our results; it seems that patients do not fully understand what care the ED provides. According to geriatric emergency department guidelines, to decrease revisits and improve quality of care for geriatric patients, the ED should improve transition care at discharge through comprehensive discharge conversations, written discharge instructions that can be understood by older patients, and a follow-up plan that includes post-discharge care [44].

### Implications for practice and research

According to older patients’ experiences, medical events leading to feelings of crises and a lower trust in the care of the GP played a role in their decision to visit the ED. After discharge, older patients experienced issues such as untreated health problems, insufficient discharge instructions, inadequate follow-up, and a lack of recovery, which may play a role in future acute care needs and new ED admissions. Given the problems identified in this study, we hypothesize that greater continuity of primary care including the identification of frailty and complex care needs, and an adequate assessment and management of symptoms by the GP or by a health care professional educated in geriatric care, may reduce the onset of crisis, and new ED admissions [36, 37]. In addition, comprehensive discharge instructions from the ED, a structured care pathway for patients with multiple chronic conditions, and hospital-at-home interventions may also reduce the onset of new crises and ED revisits [52, 53].

Moreover, we found that comprehensive discharge instructions and after care were not required for patients who were already in a specific care pathway for cancer treatment, as their home care and follow-up meetings had been pre-arranged. Geriatric patients with multiple chronic conditions may benefit from such an organized care pathway, but further research is needed to develop an effective geriatric care pathway that will reduce the number of ED revisits among older patients [46, 51, 54].

### Limitations

Patients were recruited in a university hospital, so our results might not be generalizable to patients being treated in secondary hospitals who likely have less complex conditions and other problems and perspectives. Moreover, we noticed that patients who were more fatigued by their ED visit declined to participate, so we did not obtain the perspectives of more vulnerable patients in our study. Furthermore, although all patients were interviewed within 4 weeks after their ED visit, recall bias may have occurred, i.e., patients might not have remembered all the details and experiences before their ED visit. However, we believe this effect is likely to be minimal.

## Conclusions and implications

This qualitative study identified multiple factors that may contribute to frequent ED revisits and provides insight into the perspectives and experiences of older patients. This can be useful in the development of effective interventions to reduce the need for emergency care in older patients. The identified factors included escalating feelings of crisis when symptoms increase, a poor relationship with the GP, incomplete information at discharge from the ED, and untreated, persistent health problems, inadequate follow-up and lack of recovery after an ED visit. To reduce feelings of crises and subsequent ED admissions, older patients might benefit from hospital-at-home interventions [52, 53], which can be provided by geriatric emergency teams. Identifying frailty in the ED is important for proper communication at discharge and adequate follow-up after an acute ED visit. In conclusion, our findings provide a sound basis for future studies investigating interventions to reduce the need for emergency care in older patients.

## Supplementary Information

**Additional file 1.** Interview guide.

## Data Availability

The datasets generated and analysed during the current study are not publicly available as this could potentially compromise participant privacy. Study participants consented to interviews with the understanding that their data would remain anonymous and confidential, and would not be shared beyond the researchers. The codebook generated during the analysis is available from the corresponding author on reasonable request.

## Abbreviations

ED: Emergency department
COREQ: Consolidated criteria for reporting qualitative research
MaxQDA: Computer assisted qualitative data analysis program
GP: General practitioner
