# Supplementary material for: Older patients’ perspectives on factors contributing to frequent visits to the emergency department: a qualitative interview study
Source: BMC Public Health. 2021 Sep 20;21:1709. doi: 10.1186/s12889-021-11755-z (PMC8454044; doi:10.1186/s12889-021-11755-z)
Supplement: Supplementary file 1 — Additional file 1. Interview guide. [file 12889_2021_11755_MOESM1_ESM.pdf]

Older patients' perspectives on factors contributing to frequent visits to the emergency department: a qualitative interview study

A1. Interview guide

Introduction

In the current study, we perform in-depth interviews with older adults who frequently visited the emergency department (ED) and were discharged home. After an acute visit to the ED, older adults are at high risk of revisits. Revisits would better be prevented; however, it is challenging to develop effective interventions to do so. In this study, we aim to collect the perspectives of older adults who acutely visited the ED and to explore their experiences in the period before the visit to the ED, during the ED visit, and in the recovery period after being discharged home. Using these perspectives and experiences we may identify factors that could be targeted in future developed interventions to prevent frequent visits to the ED among older adults. Therefore, we would like you to participate in this study. The interview will last approximately 90 minutes and will be voice recorded. The audio-record and transcript will only be shared within our research team. Unidentifiable parts of this interview will be published in a scientific paper.

Note: all topics need to be questioned during the interview. Bold questions are the main open-ended questions. Use probes to further clarify their thoughts. All other questions are sample questions per topic.

Topic list	*Main questions and optional further questions*
**Last acute ED visit**	• **How did you experience your last visit to the ED?**
Presenting complaint	• What was the reason you visited the ED?<br>• How was decided that you had to visit the ED?<br>• How did you feel of going to the ED at that time?
Discharge (conversation/instructions)	• **How did you experience the discharge process?**<br>• In which ways did you receive discharge information and instructions? Was it sufficient for you?<br>• How did you feel of being discharged at that time?<br>• Did you feel well prepared to be discharged home?<br>• What were your expectations of the first period after discharge?
**Period before ED visit**	• **How did you experience the period prior to your last ED visit?**

Symptoms/complaints	• Can you tell us about the course of symptoms you experienced?<br>• How did you handle those symptoms over time? How did you handle the increase in symptoms?
Functionality	• Did you experience problems in your functionality?<br>• How did you feel about this loss in functionality?
Care needs/caregivers	• How did your (informal) caregivers react on your (increasing) symptoms? What did they do for you?<br>• How did you experience the care you received prior to the ED visit?
Mental complaints	• Did you experience mental symptoms during this period?<br>• Can you tell us about your mental health in this period?<br>• Did these symptoms influence your well-being or daily activities?
**Prevention**	**• From your perspective, could there have been possibilities to prevent the acute visit to the ED?**
**Recovery after discharge**	**• How do you experience the (recovery) period after being discharged home?**
In symptoms	• How do you experience the recovery in symptoms?<br>• What do you expect from the recovery in the upcoming period?
In functionality	• How do you experience the recovery in functionality?<br>• What do you expect from the recovery in the upcoming period?
Mental complaints	• Did you experience mental symptoms after discharge?<br>• Did you experience mental symptoms related to the ED visit?
Care needs/caregivers	• Can you tell us about the care you received after discharge?<br>• How did you experience the received care?<br>• Have there been unmet care needs?<br>• Do you expect to have further care needs in the upcoming period?<br>• What do you expect from your medical doctor/general practitioner during the recovery period?

Social support	• Did you experience (sufficient) social support after discharge?<br>• How do you experience a lack of social support?
**Preventing revisits**	**• What could have been done to (further) improve your recovery in this period after discharge?**<br>**• What can be done to prevent new visits to the ED?**<br>**• Who may can help you to prevent new visits to the ED?**
